# Amelioration of Ethanol-Induced Gastric Ulcers in Rats Pretreated with Phycobiliproteins of *Arthrospira* (*Spirulina*) *Maxima*

**DOI:** 10.3390/nu10060763

**Published:** 2018-06-13

**Authors:** Oscar Guzmán-Gómez, Rosa Virginia García-Rodríguez, Lucía Quevedo-Corona, Ricardo Pérez-Pastén-Borja, Nora Lilia Rivero-Ramírez, Emmanuel Ríos-Castro, Salud Pérez-Gutiérrez, Julia Pérez-Ramos, Germán Alberto Chamorro-Cevallos

**Affiliations:** 1 Departamento de Farmacia, Escuela Nacional de Ciencias Biológicas, Instituto Politécnico Nacional, 07738 Ciudad de México, Mexico; oguz1985@live.com.mx (O.G.-G.); pastenrich@yahoo.com.mx (R.P.-P.-B.); 2 Unidad de Servicios de Apoyo en Resolución Analítica, Universidad Veracruzana, Xalapa, 91190 Veracruz, Mexico; rosga74@hotmail.com; 3 Departamento de Fisiología, Escuela Nacional de Ciencias Biológicas, Instituto Politécnico Nacional, 07738 Ciudad de México, Mexico; quevedocorona@hotmail.com; 4 Departamento de Morfología, Escuela Nacional de Ciencias Biológicas, Instituto Politécnico Nacional, 11350 Ciudad de México, Mexico; jazzband19@hotmail.com; 5 Unidad de Genómica, Proteómica y Metabolómica, LaNSE, Cinvestav-IPN, 07360 Ciudad de México, Mexico; eriosc@cinvestav.mx; 6 Departamento de Sistemas Biológicos, Universidad Autónoma Metropolitana-Xochimilco, Calzada del Hueso 1100, Col. Villa Quietud, Coyoacán, Ciudad de México 04960, Mexico; msperez@correo.xoc.uam.mx (S.P.-G.); jperez@correo.xoc.uam.mx (J.P.-R.)

**Keywords:** antiulcerogenic, *Arthrospira* (*Spirulina*) *maxima*, phycobiliproteins, ethanol

## Abstract

Phycobiliproteins of *Arthrospira* (*Spirulina*) *maxima* have attracted attention because of their potential therapeutic antioxidant properties. The aim of this study was to assess the possible antiulcerogenic activity of these phycobiliproteins (ExPhy) against ethanol-induced gastric ulcers in rats. To explore the possible mechanisms of action, we examined antioxidant defense enzymes (e.g., catalase, superoxide dismutase, and glutathione peroxidase), as well as the level of lipid peroxidation (MDA) and the histopathological changes in the gastric mucosa. Intragastric administration of ExPhy (100, 200, and 400 mg/kg body weight) significantly lowered the ulcer index value compared to the ulcer control group (*p* < 0.05). The greatest protection was provided by the concentration of 400 mg/kg. The histological study supported the observed gastroprotective activity of ExPhy, showing a reduced inflammatory response. Moreover, the alcohol-induced decrease in stomach antioxidant enzyme activity found in the ulcer control group was prevented by ExPhy pretreatment. Furthermore, ExPhy reversed the ethanol-induced increase in lipid peroxidation. In summary, the antiulcerogenic potential of ExPhy may be due, at least in part, to its anti-oxidant and anti-inflammatory effects.

## 1. Introduction

Stomach ulcers, one of the most common gastrointestinal disorders, affect people of all ages around the world [1]. Under normal conditions, the integrity of the stomach mucosal barrier is maintained by an equilibrium between irritation and defensive factors [2]. When the gastric mucosa is continuously exposed to extremely aggressive agents, such as non-steroid anti-inflammatory drugs (NSAIDs), nutritional deficiencies, smoking, stress, and excessive ingestion of ethanol, this equilibrium can be jeopardized and the risk of developing a gastric ulcer increases [3,4,5,6]. 

In the gastrointestinal tract, exposure to alcohol can damage the motility of the esophagus, stomach, and gut as well as the capacity of gut absorption. It can generate mucosal damage and even carcinogenesis [7,8]. Ethanol is a harmful agent associated with multiple pathologies and is applied orally in experimental animals to cause acute gastric lesions and ulcers [9,10]. The mechanism of ethanol-induced damage is complex and not fully understood. Ethanol produces a disruption in the integrity of the gastric mucosal barrier through exfoliation of cells, thus increasing mucosal permeability and in some cases provoking bleeding [3,11]. The extravasation of neutrophils to the site of injury triggers elevated concentrations of reactive oxygen species (ROS) and other mediators of inflammation, causing oxidative damage with deleterious effects on cells. Oxidative stress has been shown to play a role in alcohol-induced gastric mucosal damage [12,13].

*Spirulina maxima* is a blue-green alga, now given the name *Arthrospira maxima* (Am). This cyanobacterium has been used as food since antiquity, with some of the first historical records coming from the Aztec civilization and the early inhabitants of Central Africa [14,15]. Due to its high content of proteins (mainly phycocyanin and allophycocyanin), vitamins, amino acids, minerals, and essential fatty acids, it has been the object of several pharmacological studies [16]. Am has been reported as having anti-inflammatory, immunostimulatory, antiviral, and antioxidant activity [17,18,19,20], as well as producing anti-hepatotoxic and anti-nephrotoxic effects and improving vascular reactivity [21,22,23]. These effects have been related to the antioxidant activity of Am, while others are attributed to some of its active ingredients, such as phycobiliproteins, which decrease oxidative stress [19,24]. Various studies have shown that extracts of Am rich in phycobiliproteins exhibit relevant pharmacological properties, including anti-teratogenic and neuroprotective effects, antigenotoxic properties, anti-inflammatory, and antioxidant activities, and protection against colitis [19,25,26,27,28]. However, to our knowledge, there are no reports on the anti-ulcerative activities of phycobiliproteins from Am. 

Hence, the aim of the present study was to assess the gastroprotective effects of an extract of Am rich in phycobiliproteins (ExPhy) on ethanol-induced gastric ulcers in rats. Accordingly, evaluation was made of some antioxidant and oxidative markers as well as histopathological damage.

## 2. Materials and Methods

### 2.1. Preparation of the Phycobiliprotein Extract (ExPhy)

ExPhy was prepared as described by Cruz de Jesús et al. [29], with some modifications. Three grams of Am powder (AEH Spiral Spring, Mexico City) were suspended in 12 g of phosphate buffer (20 mM, pH 7) and stirred at room temperature (r.t.) for 5 min. The solution was then subjected to three cycles of freezing and thawing, being frozen at −70 °C and thawed at r.t. Subsequently, the mixture was shaken for 1 h at r.t., followed by centrifuging the crude extract of phycobiliproteins at 18,000 rpm for 30 min at 4 ° C in a Beckman Coulter Avanti j-30I centrifugue (Beckman Coulter, Brea, CA, USA). The blue supernatant obtained was separated and again centrifuged at 22,000 rpm, discarding the green precipitate after each centrifugation step. Finally, the supernatant was lyophilized and stored (protected from light) at −20 °C.

The phycobiliprotein concentration in the supernatant was calculated from absorption measurements at 562, 620, and 652 nm. Equation (1) was used for estimating C-phycocyanin (C-PC) and Equation (2) for allophycocyanin (APC) [30]:

C-PC (mg/mL) = [A620 − 0.474 (A652)]/5.34(1)

APC (mg/mL) = [A652 − 0.208 (A620)]/5.09(2)

The purity of C-PC and APC extracts was also evaluated, finding C-PC with an A620/A280 absorbance ratio and APC with an A652/A280 ratio [31].

### 2.2. LC-MALDI-MS/MS and Data Analysis

Sodium dodecyl sulfate–polyacrylamide gel electrophoresis (SDS–PAGE) was performed according to the Gallagher method [32], with a separating gel of 12% and a stacking gel of 5% acrylamide. An electrophoresis was run with 50 µg/mL ExPhy at 120 V for 90 min. Resolved proteins were visualized with Coomassie Brilliant Blue (G250) staining. Four fragments from SDS-PAGE were enzymatically digested according to the modified protocol of Shevchenko et al. [33]. The resulting tryptic peptides were concentrated at an approximate volume of 10 μL. Then, 9 μL were loaded into a ChromXP Trap Column C18-CL precolumn (Eksigent, Redwood City, CA, USA), with 350 μm × 0.5 mm, a 120 A° pore size and a 3 μm particle size, desalted with 0.1% TFA in H_2_O at a flow rate of 5 μL/min for 10 min. Then, peptides were loaded and separated on a Waters BEH130 C18 column (Waters, Milford, MA, USA), with 75 μm × 150 mm, a 130 A° pore size and a 1.7 μm particle size, using an HPLC Ekspert nanoLC 425 (Eksigent, Redwood City, CA, USA). Mobile phase A was 0.1% trifluoroacetic acid (TFA) in H_2_O and mobile phase B 0.1% TFA in acetonitrile (ACN) at a flow rate of 300 nL/min, with the following gradient: 0–3 min, 10% B (90% A); 35 min, 60% B (40% A); 36–45 min, 90% B (10% A); 46–120 min, 10% B (90% A). Eluted fractions were automatically mixed with a solution of 2 mg/mL of α-Cyano-4-hydroxycinnamic acid (CHCA) in 0.1% TFA and 50% ACN as a matrix, spotted in a stainless-steel plate of 384 spots with a MALDI Ekspot (Eksigent, Redwood City, CA, USA), with a spotting velocity of 30 s per spot at a matrix flow of 1.6 μL/min. The spots generated were analyzed by a MALDI-TOF/TOF 4800 Plus mass spectrometer (ABSciex, Framingham, MA, USA). Each MS spectrum was acquired by accumulating 1000 shots in a range of m/z 850–4000 with a laser intensity of 3100. The 100 most intense ions with a minimum signal-noise (S/N) of 20 were programmed to fragment. The MS/MS spectra were obtained after fragmentation of selected precursor ions by using collision-induced dissociation (CID), acquired by 3000 shots with a laser intensity of 3800. The MS/MS spectra were compared to the Am CS-328 database (downloaded from Uniprot, 5505 protein sequences) with Protein Pilot software v. 2.0.1 (ABSciex, Framingham, MA, USA) and Paragon algorithm [34]. Search parameters were: carbamidomethylated cysteine, trypsin as a cut enzyme, all biological modifications and amino acid substitutions set by the algorithm, and phosphorylation emphasis and gel-based ID as special factors. The detection threshold was considered at 1.3 to acquire 95% confidence, and the identified proteins observed a local FDR of 5% or less. These proteins were grouped by the ProGroup algorithm in the software to minimize redundancy.

### 2.3. Animals

Male Wistar rats (170–250 g) were supplied from the breeding colony of the Autonomous University of Hidalgo State (UAEH). The animals were maintained in cages with raised floors and wide mesh (to prevent coprophagy), in a separate animal room under standard conditions of temperature (22 ± 1 °C) and a 12 h light/dark cycle. They were fed a standard diet, with water provided ad libitum throughout the experiment. Prior to inducing ulcers, the rats were fasted for 22 h. After each experiment, the animals were euthanized in a carbon dioxide euthanasia chamber. The current protocol was accepted by the Ethics Committee of the National School of Biological Sciences (CEI-ENCB-08-2016). All procedures and handling of the animals were in accordance with the Mexican Official Regulation (NOM ZOO–062-200-1999) entitled “Technical Specifications for Production, Care, and Use of Laboratory Animals”.

### 2.4. Drugs and Chemicals

Omeprazole was acquired from Sigma-Aldrich (St. Louis, MO, USA). Thiobarbituric acid (TBA) and trichloro acetic acid (TCA) were purchased from Merck (Darmstadt, Germany). SOD and GPx were obtained from Randox, Mexico city. Other reagents and solvents, procured from local sources, were of analytical grade.

### 2.5. Antiulcer Activity and Experimental Design

The assay was carried out with the methodology described by Almasaudi et al. [35], with some modifications. The animals were randomly divided into six groups (*n* = 6). All treatments were administrated by intragastric gavage for eight consecutive days, with the gastric ulcer induced on the last day with 80% ethanol solution (1 mL/animal). Group I (vehicle control) received the vehicle only (10 mL/kg body weight (bw) of 1% Tween-80 aqueous solution). For all other groups, an ulcer was induced on the last day of treatment, one hour after administering the corresponding compound. Group II (ulcer control) was given the vehicle, group III 40 mg/kg bw omeprazole, group IV, V and VI the different concentrations of ExPhy (100, 200 and 400 mg/kg bw, respectively). 

One hour after inducing an ulcer, animals were sacrificed. The stomachs were excised, filled by injecting 2.5 mL of a 4% formaldehyde solution, and put in a beaker with formaldehyde. After 10 min, the stomachs were opened over the greater curvature and rinsed with saline solution (0.9%) to remove the blood clots. Thereafter, each gastric sample was placed on a slide. The gastric damage area (mm^2^) was determined with “Image J” image processing software. The Ulcer Index (UI) for each rat was calculated with the following formula:

UI = (TAML (mm^2^) × 100)/(TMA (mm^2^))

where TMA is the total mucosal area and TAML the total area of mucosal lesion of each rat [36]. The protection percentage (PP) was calculated using the following formula:

UI = (TAML (mm^2^) × 100)/(TMA (mm^2^))

PP = (UI control − UI treated)/(UI control) × 100

where UI control is the ulcer index of the ulcer control (group II) and UI treated is the ulcer index of the treated group (groups III–VI) [37]. From the three concentrations tested for ExPhy, the one with the least UI was adopted for all other tests.

### 2.6. Stomach Tissue Preparation

In a second experiment, another series of four groups of rats were formed. After the eight days of the corresponding treatments, the ulcer was induced and the rats were sacrificed (see previous section). The stomachs were extracted, cut along the greater curvature, and gently rinsed with cold phosphate buffer (PBS) (pH 7.4). A portion of each stomach tissue (0.5 g) was cut into small pieces and 4.5 mL of cold PBS were added. The mixture was homogenized on ice with an Ultra-turrax homogenizer (T18, IKA, Staufen im Breisgau, Germany) and a Polytron (Newtown, CT, USA) handheld homogenizer, and then tissue homogenates were centrifuged for 12 min at 12,000 rpm (4 °C). The supernatants were divided into aliquots and conserved at −20 °C until the biochemical analysis.

### 2.7. Biochemical Analysis

Gastric activity of glutathione peroxidase (GPx) was determined with a commercial kit Ransel RS504 (Crumlim, Country Antrim, UK), based on the method developed by Plagia and Valentine [38]. GPx catalyzes the oxidation of glutathione by cumene hydroperoxide. In the presence of glutathione reductase (GR) and NADPH, the oxidized glutathione is immediately converted to the reduced form with concomitant oxidation of NADPH to NADP+. The decrease in absorbance was measured after 1 and 2 min at 340 nm, with enzyme activity being directly proportional to the rate of change.

Superoxide dismutase activity (SOD) was assessed according to the method of McCord and Fridovich [39] with a Ransod SD125 Kit (Crumlim, Country Antrim, UK). The method employs xanthine and xanthine oxidase to generate superoxide radicals, which react with 2-(4-iodophenyl)-3-(4-nitrophenol)-5-phenyltetrazolium chloride (INT) to form a red formazan dye. SOD inhibits the reaction by converting the superoxide radical to oxygen. The SOD activity was determined as the degree of inhibition of this reaction, measured by absorbance at 505 nm.

Catalase activity (CAT) in gastric tissue was evaluated by tracking the rate of decomposition of H_2_O_2_ in the presence of CAT at 240 nm [40].

The protein concentration in supernatants was established by the Bradford method [41], using bovine serum albumin as a standard. This assay involves the binding of Coomassie Brilliant Blue G-250 dye to proteins at r.t. When the dye binds to the protein, it is converted from an unstable form (red in color) to a stable unprotonated form (turning blue). The blue protein dye is detected at 595 nm.

#### Lipoperoxidation Assessment

The content of malondialdehyde (MDA) was determined in each of the supernatants by the thiobarbituric acid reactive substances (TBARS) assay, as described by Esterbauer and Cheeseman [42]. To 0.5 mL of gastric mucosal homogenates were added 1.0 mL of reactive mixture containing 0.375% of TBA and 15% of trichloroacetic acid (TCA) in 0.20 N HCl. After incubation for 15 min in boiling water, the samples were cooled and centrifuged at 1000 rpm for 10 min at 4 °C. The absorbance of the supernatant was measured at 532 nm and the concentration of MDA was calculated with an extinction coefficient of 156,000 M^−1^ cm^−1^.

### 2.8. Histopathological Examination

After determination of the UI, the stomachs of each group were fixed in 10% formalin solution for 24 h. Subsequently, they were dehydrated by immersing them in ascending concentrations of alcohol solutions (70–100%) and in paraffin. Slides of stomach slices of 4–5 µm thickness were prepared and stained with hematoxylin and eosin (H&E) and then analyzed under light microscope at 20× and 40× for pathological changes, including necrosis, edema, vasocongestion, eosinophilic infiltration, and glandular damage. All slides were photographed with Zeiss Axiophot microscopy (Thornwood, NY, USA). 

### 2.9. Statistical Analysis

Statistical analysis was carried out with SigmaPlot version 12.0 (Systat Software, San Jose, CA, USA). All data are expressed as the mean ± standard error of the mean (SEM). One-way analysis of variance (ANOVA) and Dunnett’s post hoc test were applied, comparing the treated groups with the ulcer control group; statistical significance was attributed at *p* < 0.05.

## 3. Results

### 3.1. Evaluation of Phycobiliprotein Content and Purity of ExPhy

The phycobiliprotein (C-PC and APC) content and purity of ExPhy were evaluated (Table 1). C-PC purity was found to be 0.86 and APC purity 0.81. The content of C-PC was 0.40 mg/mL and of APC 0.56 mg/mL.

### 3.2. LC-MALDI-MS/MS Analysis

Liquid chromatography (HPLC) along with mass analysis by MALDI-MS/MS was carried out to identify and characterize the protein components of ExPhy, isolated from *Arthrospira maxima*. Nine different proteins were identified that belong to phycobilisomes, which is a light-harvesting macromolecular complex (Table 2). The spot number (band of gel), accession number, protein name, unused, % coverture (% Cov), and molecular weight are reported. Other proteins were also detected (their specific data are summarized in Table S1).

### 3.3. Effect of ExPhy on Ethanol-Induced Gastric Lesions

**Figure 1:**
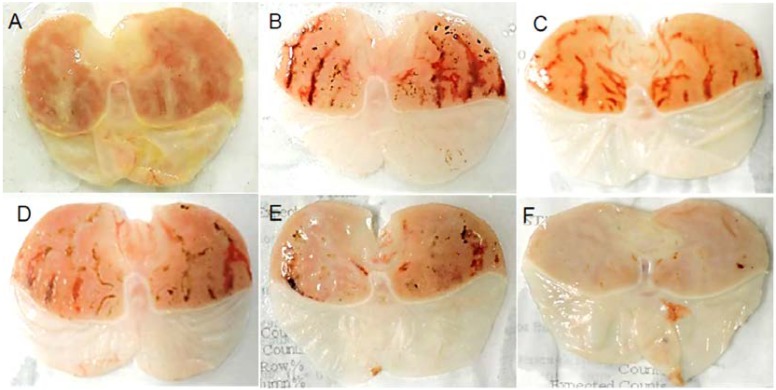
Gastric ulcer area of ethanol-induced ulceration in rats. (**A**) Vehicle control; (**B**) ulcer control; (**C**) ExPhy (100 mg/kg); (**D**) ExPhy (200 mg/kg); (**E**) ExPhy (400 mg/kg); (**F**) omeprazole (40 mg/kg).

The gastroprotective effect of pretreatment with ExPhy on ethanol-induced gastric lesions was determined (Table 3). In the vehicle control group, no macroscopic lesions were found (Figure 1A). In the ulcer control group, severe gastric lesions were observed in the mucosa layer, such as gastric hyperemia and thick linear hemorrhages (Figure 1B), with a UI of 13.73 ± 1.50. Pretreatment with ExPhy (at 100, 200, and 400 mg/kg) and omeprazole (at 40 mg/kg) (Figure 1C–F, respectively) significantly reduced the ulcer index of lesions compared to the ulcer control group, with values of 8.91 ± 0.87, 6.61 ± 1.10, 5.13 ± 0.94, and 1.32 ± 0.96, respectively. The decrease in the ulcer index was also expressed as a percentage of protection, being 35.10%, 51.87%, 62.62%, and 90.36%, respectively.

### 3.4. Histopathology

**Figure 2:**
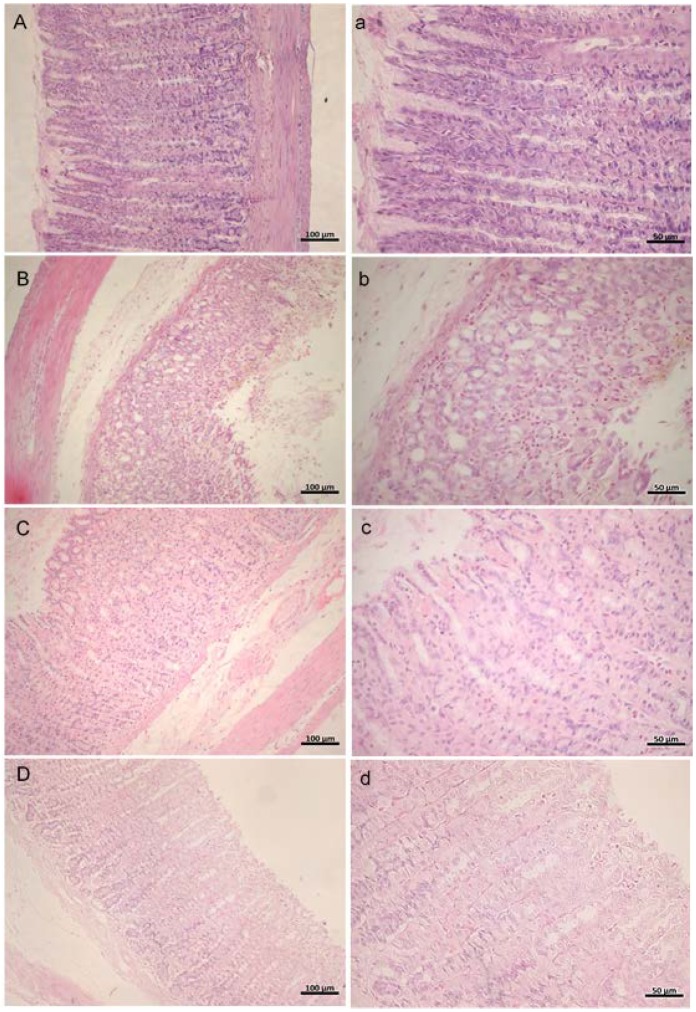
H&E staining of rat gastric mucosa in ethanol-induced gastric ulcers (magnification at 20× and 40×). (**A**,**a**) Vehicle control; (**B**,**b**) ulcer control; (**C**,**c**) omeprazole (40 mg/kg); (**D**,**d**) ExPhy (400 mg/kg).

The microscopic study of the vehicle control group (Figure 2A,a) shows typical gastric histoarchitecture with intact epithelium and glands. The ulcer control group, on the other hand, displayed several changes in the integrity of the gastric mucosa (Figure 2B,b), such as severe desquamation and loss of surface epithelial (mucous) cell, necrosis, vacuolization, edema and dilated gastric glands along with infiltration of inflammatory cells (neutrophils and eosinophils). Pretreatment with omeprazole decreased the gastric lesions compared to the ulcer control. The gastric mucosa exhibited focal loss of superficial gastric epithelium. The gastric glands were almost normal in appearance. There was mild edema with limited eosinophilic infiltration and minimal hemorrhage (Figure 2C,c). Pretreatment with ExPhy resulted in gastric lesions, characterized by focal areas of disruption in one-third of the mucosa, without a mucus layer in this zone. Nevertheless, the rest of the mucosa showed almost normal gastric glands, with mild edema and limited eosinophilic infiltration (Figure 2D,d) compared to the ulcer control.

### 3.5. MDA and Antioxidant Enzyme Determination 

**Figure 3:**
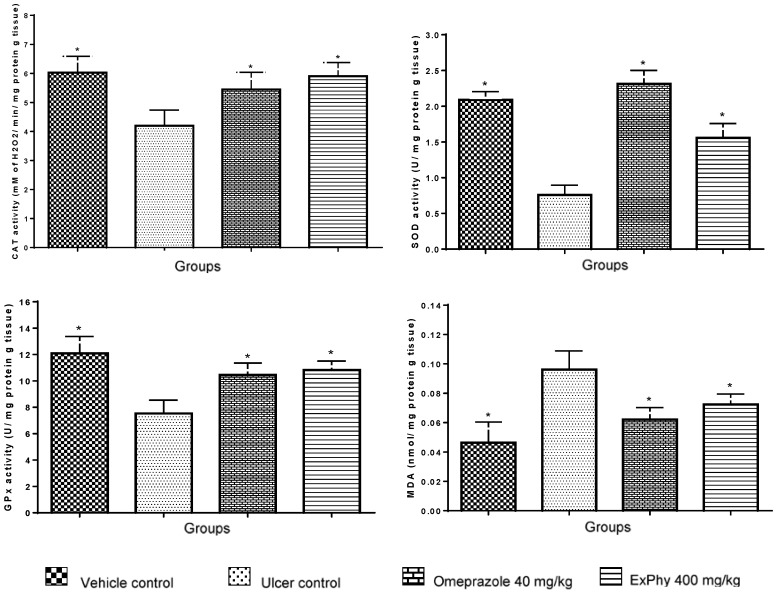
ExPhy and omeprazole pretreatments, followed by ethanol-induced gastric ulcers, produced protective effects on the gastric mucosal activity of GPx, SOD and CAT, as well as lowering the levels of MDA compared to the ulcer control. * Indicates *p* < 0.05. Data are expressed as the mean ± SEM. ExPhy, extract rich in phycobiliproteins.

After ethanol administration, an evaluation was made of the effect of ExPhy on the activity of antioxidant enzymes (SOD, CAT, and GPx) and the level of MDA (as a lipoperoxidation index) in gastric tissue (Figure 3). The SOD enzyme activity in the ulcer control significantly decreased compared to the vehicle control. Pretreatment of rats with ExPhy (400 mg/kg) and omeprazole (40 mg/kg) significantly restored SOD activity in relation to the ulcer control. CAT activity in the stomach of the ulcer control was significantly lower than that of the vehicle control. In the groups treated with ExPhy (400 mg/kg) and omeprazole (40 mg/kg), CAT activity was significantly greater than in the ulcer control. The depletion of GPx activity observed in ulcer control was significantly reversed in rats pretreated with ExPhy (400 mg/kg) and omeprazole (40 mg/kg). Regarding gastric MDA, there was a significantly higher level in the ulcer control versus the vehicle control. Pretreatment with ExPhy (400 mg/kg) and omeprazole (40 mg/kg) protected against the damage found in the ulcer control and led to decreased concentrations of MDA. 

## 4. Discussion

Considering the frequency of gastric ulcers in humans and the side effects and cost of some available synthetic drugs, the use of natural products represents an important alternative for many [43,44]. In this sense, *Spirulina maxima* and ExPhy have proven to be advantageous in the treatment of various ailments in lab animals and patients. Moreover, their absence of toxicity has been demonstrated by short- and long-term studies [45].

The current investigation evaluated the antiulcerogenic activity of ExPhy of Am on an ethanol-induced gastric ulcer model. A determination was made of the effects of ExPhy in relation to some antioxidant and oxidative markers, along with protection against histopathological damage. C-PC and APC in ExPhy were identified and characterized by standard analytical methods (UV–VIS spectroscopy and MALDI-MS/MS).

Phycobilisomes are supramolecular complexes on the stromal surface of the thylakoid membrane in cyanobacteria (e.g., Am). These complexes, which participate in trapping light energy and transferring it within the cell, can make up to 60% of the total protein [46,47]. The antioxidant potential reported for Am may be attributed to this major class of proteins. Phycobilisomes are constituted mainly by individual protein components denominated phycobiliproteins and linker polypeptides [48]. Phycobiliproteins consist of two different polypeptides (the α and β chains) that are covalently linked to bilin chromophores [49]. In this study, MALDI-MS/MS analysis corroborated that the protein bands excised from SDS-PAGE belonged to α and β subunits of C-PC and APC, the main photosynthetic accessory pigments present in cyanobacteria [47]. On the other hand, the values of purity achieved for C-PC and APC from ExPhy (Table 1) can be considered good, since a purity of 0.7 is accepted as food grade [50]. Interestingly, the analysis by mass spectrometry showed the presence of other cellular proteins (see the Supplementary Materials) that probably influenced the purity of phycobiliproteins in a minor way. 

Phycobiliproteins have attracted attention due to their special structure and potential therapeutic properties, either in a pure state or in protein extract. C-PC and APC are known to exert several beneficial activities, including antioxidant (shown in vitro and in vivo) [19,51,52], anti-inflammatory, and immune-stimulatory [27,53,54,55,56]. The antioxidant mechanism of phycobiliproteins has been associated with various activities: (1) ROS scavenging [25,51,52]; (2) chelating [25]; (3) neutralizing free radicals through the sulfur atom of cysteine and methionine of apophycocyanin, which can transfer hydrogen atoms or electrons to free radicals [51,57]; and (4) influencing the activity of antioxidant enzymes [58,59,60].

Excessive ethanol consumption is considered one of the risk factors for gastric ulcers in humans [61,62]. Its use in experimental animals allows for the evaluation of cytoprotective activity of potentially active products [63]. Different mechanisms of gastric cytoprotection have been suggested, including increased gastric mucosal blood flow, free radical scavenging, and stimulation of cell growth and repair [64]. In the current study, consistent with previous findings, administration of 80% ethanol solution by intragastric gavage produced marked damage in the gastric mucosa of rats, characterized mainly by elongated macroscopic lesions with intense hemorrhaging and hyperemia, as well as loss of mucus [35,65,66]. Pre-treatment of rats for eight days with ExPhy markedly attenuated gastric damage and promoted healing of gastric mucosa lesions induced by ethanol, although to a lesser extent than the standard drug, omeprazole. ExPhy provided the best protection at the highest concentration tested. These results indicate that ExPhy may have a protective effect against the ulcerative lesions induced by ethanol on gastric mucosa. 

Additionally, though it was not explored presently, direct contact of phycocyanin with injured gastric mucosa possibly contributes to the healing process. There is evidence that both Spirulina and C-phycocyanin are capable of stimulating cell growth and viability, both in human keratinocytes and in a rat model [67]. These properties underlie the use of Spirulina in the development of new biomaterials for the construction of scaffolds for cell growth in the field of tissue engineering [68]. 

After demonstrating that ExPhy provided a protective effect against the development of ethanol-induced ulcers, the next step was to confirm these findings through a histopathological analysis of gastric tissue. In accordance with previous studies, the ulcer control group showed typical histological damage 1 h after ethanol administration. This damage was characterized by vascular congestion, submucosal edema formation, loss of gastric mucosa integrity, and necrotic tissue injury, as well as an inflammatory response characterized by neutrophil and eosinophil infiltration [65,69,70]. The aggregation of neutrophils plays a fundamental role in the process of injury and inflammation in the gastric mucosa due to their release of tissue-disruptive substances like proteases, leukotrienes B4 (LTB4), and reactive oxygen species [71,72]. Via NADPH oxidase, neutrophils release superoxide anions, and these in turn are metabolized into the hydroxyl radical. The latter can mediate lipid peroxidation of polyunsaturated fatty acids and cause damage to cell membranes, leading to an alteration in the structural integrity and biochemical function of membranes [73,74]. 

Interestingly, the microscopic study revealed a lesser extent of inflammatory infiltrate in the group of rats treated with ExPhy. Moreover, the histopathological changes triggered by ethanol were significantly diminished. The gastric mucosa showed a more regular architecture and less hemorrhaging and submucosal edema. Previous reports have confirmed the anti-inflammatory properties of phycobiliproteins. In rats with colitis treated with phycocyanin, Gonzales et al. [27] described a substantial reduction in neutrophil infiltration in colonic mucosal injury. Remirez et al. [28] evaluated the protective effect of the phycocyanin extract in the zymosan-induced arthritis model in mice, finding that treatment with phycocyanin displayed an inhibition of cellular infiltration. Further studies carried out by Romay et al. [75,76] demonstrated that phycocyanin is able to inhibit the inflammatory response and edema triggered by 12-O tetradecanoyl phorbol 13-acetate in mice, as well as reduce the LTB4 levels in arachidonic acid-induced mouse ear edema. Inhibitors of cyclooxygenase (COX) and lipooxygenase (LOX) enzymes have proven to be active in this model [77], suggesting that the mechanism of action of ExPhy for diminishing inflammatory infiltrate and edema could be linked, at least in part, to their antioxidant properties (as previously described), an inhibitory effect on cyclooxygenase-2 (COX-2), and/or the biosynthesis of LTB4.

Currently, there is consensus that alcohol intake is noxious to gastric tissue. The generation of ROS and subsequent oxidative stress is one of the major mechanisms in the pathogenesis of gastric tissue damage and ulcerogenesis induced by ethanol [71,78]. It has been documented that the administration of alcohol not only has necrotizing effects but also gives rise to oxidative stress by provoking injury to the mitochondria. The latter occurs through a decrease in mitochondrial membrane potential, which leads to a perturbation of the mitochondrial electron transfer system and an overproduction of O_2_—[79,80]. Oxidative stress is manifested as an abnormal elevation of reactive oxygen species, leading to the depletion of the antioxidant defense system (enzymatic and non-enzymatic), thus furthering damage to cell structures such as carbohydrates, nucleic acids, proteins and lipids (promoting lipid peroxidation) [12,81]. Potent antioxidants and free radical scavengers have been shown to inhibit oxidative stress and consequently the progression of lipid peroxidation [82,83]. Molecules with this capability include flavonoids, phenolic compounds, vitamins (tocopherol), and phycocyanin [53]. The latter is a powerful antioxidant that removes free radicals, including peroxynitrite radicals, nitric oxide radicals, peroxyl radicals, hydroxyl radicals, superoxide anion, hypochlorous oxygen, hydrogen peroxide, and synthetic radicals DPPH and ABTS. This action is given by its structure, rich in amino acids such as methionine, cysteine, and the tetrapyrolic prosthetic group, which can stabilize highly reactive species such as free radicals [60]. In addition, in vivo and in vitro models have been shown to exert antioxidant action within the cells [84]. Therefore, the administration of ExPhy in this study probably improves cellular antioxidant defenses.

In the current study, we corroborated that intragastric administration of ethanol causes severe oxidative stress in stomach tissue (ulcer control group) by significant inhibition of the activity of antioxidant enzymes such as CAT, GPx, and SOD compared to the vehicle control group. Additionally, there was a significant increase in the level of MDA, as previously reported [66,85]. MDA is commonly measured as a biomarker to assess lipid peroxidation levels in tissues [86]. ExPhy pretreatment exhibited antioxidant properties by decreasing the levels of MDA, suggesting its potential to protect against ethanol-induced lipid peroxidation in rats. Furthermore, ExPhy preserved the antioxidant activity of GPx, CAT, and SOD enzymes after ethanol administration, thus protecting the gastric mucosa. 

Normally, these antioxidant enzymes provide cells with mechanisms for defending themselves against ROS damage. SOD represents the first line of defense against ROS by catalyzing the conversion of O_2_—to oxygen and H_2_O_2_, the latter of which is catalyzed to H_2_O by CAT or GPx [83]. The possibility of this protective effect being fostered by ExPhy is consistent with previous findings that phycobiliproteins engender a significant decrease in oxidative stress by increasing the antioxidant defense system and reducing the levels of lipid peroxidation in different pathologic conditions. Fernandez-Rojas et al. [60] reported the protective effect of C-PC against cisplatin-induced nephrotoxicity in CD-1 mice through the attenuation of oxidative stress and an enhancement of the activity of antioxidant enzymes. This effect was associated with the ROS-scavenging ability of C-PC. Additionally, Rodríguez-Sánchez et al. [87] found that phycobiliproteins protect renal cells against mercury-induced oxidative stress in mice. The mechanism of action suggested involves the reduction of oxidative markers and the chelating properties of phycobiliproteins. More recently, Kumari and Anbarusa [59] documented the protective action of C-PC in the rat selenite cataract model, which might be a consequence of its ability to scavenge the free radicals generated and exert an anti-apoptotic function.

In conclusion, the current results suggest a significant gastroprotective effect of ExPhy against ethanol-induced gastric damage. This protection may be related to the antioxidant properties of ExPhy by activating some enzymatic antioxidant mechanisms (SOD, CAT, and GPx), diminishing lipid peroxidation, and attenuating the inflammatory response, improving defenses against the erosive lesion that characterize the development of gastric ulcers produced by ethanol. However, further detailed studies are needed to clarify the mechanisms underlying the gastroprotective effect shown by ExPhy. 

## Figures and Tables

**Table 1:** Concentration and purity ratio of ExPhy.

Phycobiliprotein	Concentration (mg/mL)	Purity Ratio A620/A280 (C-PC) A652/A280 (APC)
C-PC	0.40	0.86
APC	0.56	0.81

C-PC, Phycocyanin C; APC, Allophycocyanin; ExPhy, extract rich in phycobiliproteins.

**Table 2:** Results of different protein spots identified by MALDI-MS/MS.

No.	Spot No.	Accession	Protein	Unused	% Cov	MW (Da)
1	1, 2, 3, 4	tr|Q8VRJ2	Phycocyanin alpha chain	16	69.75309	17,600
2	1, 2, 3, 4	tr|Q7BA94	Phycocyanin beta chain	10.44	78.48837	18,094
3	1, 2, 3, 4	tr|B5VUA2	Allophycocyanin, beta subunit	8	82.60869	17,330
4	1, 3, 4	tr|B5W3K3	Allophycocyanin, beta subunit	4.85	56.80473	18,442
5	1, 2, 3, 4	tr|B5W789	Phycobilisome linker polypeptide	2.8	52.7559	29,427
6	1, 2, 3	tr|B5VV49	Phycobilisome linker polypeptide	2.75	30.90278	32,509
7	2	tr|B5VV50	Phycobilisome linker polypeptide	3.67	59.77859	30,834
8	3, 4	tr|B5W2H7	Phycobilisome protein	8.72	55.90062	18,002
9	1, 2, 3, 4	tr|B5VUA1	Phycobilisome protein	9.49	80.12422	17,392

No, number; % Cov, % coverture; MW (Da), molecular weight (daltons); MALDI-MS/MS, matrix-assisted laser desorption/ionization mass spectrometry.

**Table 3:** Effect of ExPhy and omeprazole on ulcer parameters in rats with ethanol-induced ulcers.

Groups	Pretreatment	Ulcer Index (mm^2^)	Protection Percentage (%)
I	Vehicle control	0 *	0
II	Ulcer control	13.73 ± 1.50	0
III	Omeprazole (40 mg/kg)	1.32 ± 0.96 *	90.36
IV	ExPhy (100 mg/kg)	8.91 ± 0.87 *	35.10
V	ExPhy (200 mg/kg)	6.61 ± 1.10 *	51.87
VI	ExPhy (400 mg/kg)	5.13 ± 0.94 *	62.62

Data are expressed as the mean ± SEM; *n =* 6 rats per group; * indicates *p* < 0.05 compared to the ulcer control group; ExPhy *=* extract rich in phycobiliproteins.

## Supplementary Materials

The following are available online at http://www.mdpi.com/2072-6643/10/6/763/s1, Table S1: Additional proteins identified by MALDI-MS/MS.
